# Potential Role of Masting by Introduced Bamboos in Deer Mice (*Peromyscus maniculatus*) Population Irruptions Holds Public Health Consequences

**DOI:** 10.1371/journal.pone.0124419

**Published:** 2015-04-21

**Authors:** Melissa C. Smith, Richard Gomulkiewicz, Richard N. Mack

**Affiliations:** 1 USDA-ARS, Invasive Plant Research Laboratory, 3225 College Ave, Fort Lauderdale, Florida, United States of America; 2 Washington State University, School of Biological Sciences, Pullman, Washington, United States of America

## Abstract

We hypothesized that the ongoing naturalization of frost/shade tolerant Asian bamboos in North America could cause environmental consequences involving introduced bamboos, native rodents and ultimately humans. More specifically, we asked whether the eventual masting by an abundant leptomorphic (“running”) bamboo within Pacific Northwest coniferous forests could produce a temporary surfeit of food capable of driving a population irruption of a common native seed predator, the deer mouse (*Peromyscus maniculatus*), a hantavirus carrier. Single-choice and cafeteria-style feeding trials were conducted for deer mice with seeds of two bamboo species (*Bambusa distegia* and *Yushania brevipaniculata*), wheat, *Pinus ponderosa*, and native mixed diets compared to rodent laboratory feed. Adult deer mice consumed bamboo seeds as readily as they consumed native seeds. In the cafeteria-style feeding trials, *Y*. *brevipaniculata* seeds were consumed at the same rate as native seeds but more frequently than wheat seeds or rodent laboratory feed. Females produced a median litter of 4 pups on a bamboo diet. Given the ability of deer mice to reproduce frequently whenever food is abundant, we employed our feeding trial results in a modified Rosenzweig-MacArthur consumer-resource model to project the population-level response of deer mice to a suddenly available/rapidly depleted supply of bamboo seeds. The simulations predict rodent population irruptions and declines similar to reported cycles involving Asian and South American rodents but unprecedented in deer mice. Following depletion of a mast seed supply, the incidence of Sin Nombre Virus (SNV) transmission to humans could subsequently rise with dispersal of the peridomestic deer mice into nearby human settlements seeking food.

## Introduction

A plant invasion can trigger a concatenation of ecological consequences within the new range by creating, enhancing/dampening or eliminating species’ interactions in ways the native biota cannot e.g., through introduction of a N-fixing plant into a range with chronically low soil N [1–3], creation of an arboreal canopy in a heretofore treeless community [4,5] or habitat alteration conducive to a parasite-carrying insect [6]. Novel consequences could also arise if the invader were to provide a highly palatable, nutritious food subsidy that boosts population growth rates for an opportunistic native predator [7]. An invader that periodically and rapidly augments the food supply for consumers, i.e., a pulsed resource event (*sensu* Ostfeld & Keesing [8]), could amplify species’ responses beyond those produced by native non-masting species.

Many temperate bamboos reproduce in species-wide masting events, wherein all individuals simultaneously flower and set seeds across a wide range and then die [9–11]. In temperate forests in South America and eastern Asia, the synchronous maturation of bamboo seeds provides a pulsed resource that in short order attracts rodents that gorge upon the temporarily plentiful seed crop [9,12,13]. Although predation may restrict the size of seed predator’s populations at low density, predators have little effect when their prey is not food-limited [14]. Consequently, the rodent population undergoes a rapid increase [12,15,16]. After exhausting the transient seed supply, the starving animals disperse in search of food, even attacking crops and invading dwellings [13,17,18].

Such a surge in the size of a peridomestic rodent population can trigger a consequence far more serious than crop destruction—an increase in the frequency of transmission of rodent-borne pathogens to humans. For example, masting bamboos in temperate South American forests have been implicated in rapid population outbreaks among granivorous rodents that serve as reservoirs for human pathogens [12]. In each of these South American scenarios, a masting bamboo is the source of the pulsed resource (seeds) that initiates the series of events, ultimately resulting in a spike in the number of human infections.

Coniferous forests in western North America are physiognomically similar to these South American forests as well as coniferous forests in eastern Asia but they lack native bamboos [19]. The absence of bamboos in coniferous forests in western North America may however change. Growing horticultural interest in ornamental bamboos has accelerated bamboo importation into the U.S., including the West Coast [20]. As a result, a growing list of Asian bamboos are now naturalized in the U.S. (e.g., *Phyllostachys aurea*, *P*. *aureosulcata*, *P*. *nigra*, *Pseudosasa japonica*) (http://www.eddmaps.org, accessed 20 October, 2014), including species with pronounced masting cycles [9]. Increasingly prominent among these introductions are leptomorphic, i.e., running, bamboos (e.g., *Phyllostachys* spp., *Sasa* spp.) that tolerate prolonged frost and snow cover [21], intense shade [22] and spread by vigorously growing clones [23]. These naturalizations have occurred at multiple locations in California (*Phyllostachys aurea)* and the Pacific Northwest of the U.S. (hereafter PNW) (*Pseudosasa japonica*) (http://www.eddmaps.org, MCS, unpublished data), thereby placing extensive western coniferous forests well within these species’ potential new ranges. Masting events in each bamboo species is triggered by intrinsic, rather than extrinsic factors, such that all individuals, regardless of location mast simultaneously, including those in North America [9]. For example, all observed individuals of *Phyllostachys aurea*, *Bambusa textilis* and *Fargesia murilae* flowered simultaneously in native Chinese communities and cultivated sites in North America and Europe (see S1 Table).

Could the naturalization and potential invasion of Asian bamboos in North American forests produce food subsidy-induced surges in rodent populations analogous to cases repeatedly reported in Asia and South America [12,15]? Requisite for such a surge would be the occurrence of an abundant granivorous rodent in these forests. *Peromyscus maniculatus* (deer mouse), a widespread native rodent in many western North American forests and adjacent grasslands, is a voracious omnivore (e.g. arthropods, seeds, fungi) [24,25] that also readily attacks untreated grain seed. Furthermore, these rodents will migrate to a pulsed resource and display a marked increase in fitness, including an extension of reproduction into winter [26–28].

Similar to rodents elsewhere, deer mice are also a reservoir of human pathogens, including Sin Nombre Virus (SNV) [29], *Francisella tularensis* subsp. *holartica* (Tularemia) [30], and *Borrelia burgdorferi* (Lyme disease, transmitted by *Ixodes pacificus*) [31–33]. Sin Nombre Virus, the primary cause of hantavirus pulmonary syndrome, is an especially serious health threat with an average mortality rate of 37% mortality rate in the United States [34]. Seroprevalance, the proportion of individuals carrying viral antibodies, can rise when deer mice populations reach high densities [35]. During population booms of deer mice, humans are at increased risk of encountering an infected animal in peridomestic settings and dwellings: seropositive mice in Oregon and Montana ranged from 0–68% of the population, and 121 cases of SNV have been reported in Idaho, Montana, Oregon and Washington since 1993 [7,36,37].

We asked whether synchronous flowering/fruiting by a naturalized bamboo population in PNW forests could cause a feast and famine response in deer mice populations similar to the response of bamboo seed-feeding rodents in Asia and South America [13,15,38], thereby increasing the intensity of seed predation by deer mice as well as the opportunity for transmission of human pathogens. We addressed through experimentation and modeling three questions about the potential foraging of *P*. *maniculatus* on Asian bamboo seeds. 1) Can deer mice survive, i.e., maintain weight, on a diet comprised solely of bamboo seeds? 2) To what extent can deer mice reproduce solely on a diet of bamboo seeds? 3) What demographic consequences ensue for a deer mouse population gorging on the temporarily abundant seeds produced by masting populations?

## Materials and Methods

### Ethics Statement

All protocols involving research animals were conducted with strict adherence to guidelines set by the National Research Council’s Guidelines to the Care and Use of Laboratory Animals (3^rd^ Ed.) and were approved by Washington State University’s Institutional Animal Care and Use Committee under the protocols #03821–001 and #03885–002.

### Feeding Trials

All deer mice in this study were obtained from the Peromyscus Genetic Stock Center (University of South Carolina, Columbia, SC). Feeding trials tested seeds of two Asian bamboo species (*Bambusa distegia* and *Yushania brevipaniculata*), wheat (*Avena*), *Pinus ponderosa*, a standard laboratory rodent feed (Harlan Teklad rodent diet, Harlan Laboratories, Indianapolis, IN) and a native mixed diet based on stomach-contents from deer mice collected in regional forests [39,40]: 40% forb *(Balsamorhiza sagittata)* and 50% grass (*Pseudoroegnaria spicata)* seeds mixed with insect larvae, *Chilecomadia moorei* and *Tenebria molitor* (5% each, 10% total). We procured wheat seeds in eastern Washington; *B*. *sagittata*, *P*. *spicata*, and *P*. *ponderosa* seeds were field collected from the George Hudson Biological Reserve (Whitman Co., WA) and the Forest Nursery (Potlatch, ID). Bamboo seeds (*B*. *distegia* and *Y*. *brevipaniculata)* were produced in 2008 and 2009 in Sichuan, People’s Republic of China (PRC) during two large masting events (SINA News Corp 2008, http://news.sina.com.cn/c/2008-03-24/040315209174.shtml) and were obtained from Yunnan Rumaoda Investment Co., LTD, Kunming, PRC. We were unable to locate seeds from any naturalized species in the U.S. because no recent masts have occurred. However, the seeds we obtained approximate the seed length (3–5 mm) and form (dry caryopses) of other temperate leptomorphic bamboos and did not vary nutritionally. Live insect larvae were obtained from Fluker Farms (Port Allen, LA). Caloric content of each food item is reported in S3 Table.

The first feeding trial examined the outcome for animals on a single-choice diet. Same-sex paired mice were kept in standard polycarbonate cages (8 cm x 12.5 cm x 5.5 cm) with nesting material to provide environmental enrichment and reduce stressors [41].

Cages were randomly assigned to 28-day feeding trials with cage as the experimental unit (N = 8 cages per feed type) and shelf as the blocking factor. Cages received 16 g of food per day (8 g/animal); uneaten food was removed daily. Mice were individually weighed every two days. Two male mice (from separate cages) died during the second week of the study. Based on the necropsies and histopathology reports conducted by the Washington Animal Disease Diagnostic Laboratory, Washington State University, Pullman, WA, the deaths were ascribed to non-specific natural causes, with no clear cause of death. Neither animal displayed unhealthy tissues or lesions. Changes in combined and individual weights of the animals were analyzed using a repeated measures ANOVA in a linear mixed-effects model (package [nlme] in R [42]), although we only report the combined cage weight here because individual mouse weights within the cage are not independent.

Multiple-choice feeding trials were conducted in single-mouse cages. Mice were allowed to openly feed for 12 h beginning at 20:00 and ending at 08:00 with 5 g of each of the food choices listed above (Note: the native mixed diet was comprised of 50% forbs and 50% grass by volume, and bamboo seeds in this trial were restricted to *Y*. *brevipaniculata*). Uneaten food and seed cast-offs were then collected, sorted and weighed. Six feeding trials were conducted with 10 mice each; a minimum of 24 h elapsed between feeding trials and the same 10 mice were used for each trial. These data were evaluated with multivariate analyses because food choices presented within the trial are not considered independent [43,44]. To account for potentially confounding effects of food choice based on factors other than availability, we standardized trials by analyzing the proportion of each food choice within the whole diet. We then pooled data for each mouse (N = 10) and used Hotelling's *T* ^*2*^ approach, as described in Lockwood [43], within the R software package (version 3.0.2, Package ‘Hotelling’, [45]) to analyze proportional feeding choices.

Effects of single-choice diets on reproduction and pup weight at weaning were estimated by randomly assigning females to one of six diets and housing them individually in cages arranged in a completely randomized block design (shelf as the blocking factor, N = 10). We conducted this procedure with naïve females and females that had first reared a litter while consuming laboratory rodent feed before initiating the single-choice feeding trial. Rearing experience had no effect on conception rates, litter size or rearing success (χ^2^, P > 0.09 for all responses); therefore, we pooled data from both experienced and naïve females. Each female received 8 g of the assigned diet for 10 days before a male was added to the cage. Males were paired with females for 10 days during which both animals received the assigned diet. After males were removed, feed was increased to 16 g per female, and females were weighed daily to detect impregnation. Uneaten food was removed daily. Nursing deer mice may cannibalize young when handled, so pups were not weighed at birth. Lactating females were maintained on a 16 g per day diet for 24 days after producing pups; pups were then weighed and weaned. Several pups died from cannibalization (no statistical difference between treatments, ANOVA, P >0.31) and a one-time laboratory accident. Cannibalization of deer mice young in laboratory settings is common and has multiple causes [46]. The fate of these mice was not included in the statistical analyses. The fate of these mice was not included in the statistical analyses. All counts used in the statistical analysis are pups that survived to weaning; these animals were sexually mature when 42 days old (approximately two weeks after weaning). We analyzed pups per litter using the Kruskal-Wallis non-parametric test (due to violations of normality assumptions). Pup weight at weaning was analyzed using a one-way ANOVA within the R software package [42]. Shelf (block) was ruled out as a factor using a two-way ANOVA for both pups per litter and pup weight (P > 0.27). Cochran’s Q was performed using PROC FREQ within SAS (version 9.3) to compare rates of conception among diets.

### Parameterizing the Mathematical Model of Bamboo Seed-Deer mouse Feeding Dynamics

Data from the reproduction trials were used to parameterize a mathematical model describing the joint dynamics of bamboo seeds and deer mice densities after a masting event. Pines and oaks also undergo masting events that incur seed predation [47,48]; data from these native examples were also used to parameterize comparative models. We used a modified version of the Rosenzweig-MacArthur consumer-resource model [49] ([50] for additional background on the model and its parameters) (Table 1, see S2 Table for value derivations). The dynamics of the masting seeds are described by the differential equation

$$\frac{dB}{dt}=-a\frac{MB}{h+b}-\rho B$$ (Eq 1)

where *B* = *B*(*t*) is the average energy available per hectare based on the average seed values [51–54], *Pinus ponderosa* [48], and North American *Quercus* spp. [55] *t* weeks after a masting event, and *M* = *M*(*t*) is the mouse density at week *t* (individuals per hectare [56,57]). All seed densities were converted to kJ per ha to account for differences in relative seed size and caloric content (i.e. fewer, larger seeds are weighted differently in a model than many small seeds). The maximum per mouse seed attack rate is *a* (measured in kJ per ha per week), and *h* is the seed density (kJ per ha) at which the per mouse attack is *a*/2, i.e., half the maximum. We estimated *h* by back-calculating from the average length of the mast event for each taxon (oaks, 3 months; pines, 3 months; bamboo, 12 months; see supporting information). Rate of seed removal by other sources including fungal attack and germination is symbolized by *ρ*. Deer mice dynamics are described by the differential equation

$$\frac{dM}{dt}=\left[\beta +ca\frac{B}{h+B}\right]M-\left[\delta +\frac{M}{K(B)}\right]M+i(B)$$ (Eq 2)

where parameter *β* is background per capita mouse birthrate (i.e., rate in the absence of seeds), *c* is a conversion factor for seed density consumed to mice density produced, *δ* is background per capita mouse death rate and *i(B)* is an immigration term (individuals arriving per ha per week). The term *K*(*B*), the functional carrying capacity of mice on a given seed diet, is inversely related to the strength of intraspecific density regulation in deer mice, which may depend on the seed availability (*B*). We included an immigration term *i*(*B*) based on observations from other population irruption events [57] in which 30 new individuals enter the population every week as long as *B*_*threshold*_ *< B*, where *B*_*threshold*_ is the energetic value in seeds (in kJ) required to attract immigrants. We estimated this value at 3000 kJ based on per capita consumption rates. Once *B < B*_*threshold*_, immigration ceases. That is, *i*(*B*) = 30 for *B* > 3000 and *i*(*B*) = 0 for *B* ≤ 3000.

**Table 1:** Model parameter variables and values.

Definition	Variable	Parameter Value	Units	Source
Maximum attack rate (bamboo)	*a*_*bamboo*_	0.009	kJ ind^-1^ha^-1^wk^-1^	Calculated from measured mouse daily consumption rates
Seed density at attack rate half saturation	*h*_*bamboo*_	322	kJ ha^-1^	See supporting information for calculations
Maximum attack rate (oak)	*a*_*oak*_	0.009	kJ ind^-1^ha^-1^wk^-1^	
Seed density at attack rate half saturation	*h*_*oak*_	71431	kJ ha^-1^	
Maximum attack rate (pine)	*a*_*pine*_	0.009	kJ ind^-1^ha^-1^wk^-1^	
Seed density at attack rate half saturation	*h*_*pine*_	78835	kJ ha^-1^	
Other sources of seed removal	*ρ*	0.25	Percentage of seeds removed ha^-1^wk^-1^	Estimated from Crawley 1989
Seed to mouse conversion factor	*c*	205	offspring kJ^-1^female^-1^	Calculated from measured mouse daily consumption rates
Background mouse birth rate	*β*	0.07	offspring female^-1^wk^-1^	French & Kaaz 1968 (daily rate)
Background mouse death rate	*δ*	0.02	individuals wk^-1^	French & Kaaz 1968 (daily rate)
Initial mouse density	*M*_*0*_	(*β-δ*)*K_min_	individuals ha^-1^	equilibrium in absence of bamboo seeds
Initial bamboo density	*B*_*0*_	73,482,500	kj ha^-1^	Calculated from mean observed seed-fall
Initial oak density		4,362,187	kj ha^-1^	
Initial pine density		4,814,400	kj ha^-1^	
Mouse carrying capacity	*K*_*min*_	120	individuals ha^-1^	Background mouse carrying capacity

In general, *K*(*B*) should increase with *B*, indicating weaker intraspecific competition as seed abundance increases. We utilized a logarithmic equation for *K(B)* for the results below,

$$K(B)=K_{\min}+\ln(1+B)$$ (Eq 3)

based on the reproductive biology of *P*. *maniculatus* [58]. The population dynamics are, however, qualitatively similar when *K*(*B*) increases linearly or even exponentially from *K*_min_ (the pre-mast mouse carrying capacity) when *B* = 0. Our projections are then qualitatively robust for any monotonic increasing relationship between intraspecific competition and seed density.

Masting events are fundamentally transitory and produce abundant seeds in a very short period [9]. Consequently we assume a high density of bamboo seeds is present at *t* = 0, *B*_0_, the time of the masting event. In turn, we assume the initial density of deer mice is at equilibrium before the onset of masting, when *B* = 0. This initial density can be found by setting *dM*/*dt* = 0 and *B* = 0 in Eq (2) and solving for *M* giving:

$$M_{0}=(\beta -\delta )+K_{\min}$$ (Eq 4)

We solved the system of differential Eq (1) and (2) with initial conditions *B*(0) = *B*_0_ and *M*(0) = *M*_0_ (Eq 4), numerically in the programming language R for Mac OS X, version 1.65 [42] using the library ‘desolve’. All values used to parameterize the model were extrapolated from field studies (*B*, *K*, *M*, *β*, *δ*) [54,57–60] or calculated and measured from our experiments (*c*, *a*, *h*, *ρ*) [61–63] (Table 1).

## Results

We initially evaluated the ability of *P*. *maniculatus* to survive and reproduce solely on a diet of seeds of the bamboos, *B*. *distegia* or *Y*. *brevipaniculata*, in laboratory feeding trials. We compared weight change in all mice in single-choice feeding treatments (laboratory rodent feed, *P*. *ponderosa*, *Avena* spp., *B*. *distegia*, *Y*. *brevipaniculata* seeds and native mixed diet). All animals maintained weight on all diets throughout the feeding trials (ANOVA, F_7, 48 = 0.637_, P = 0.772).

**Fig 1:**
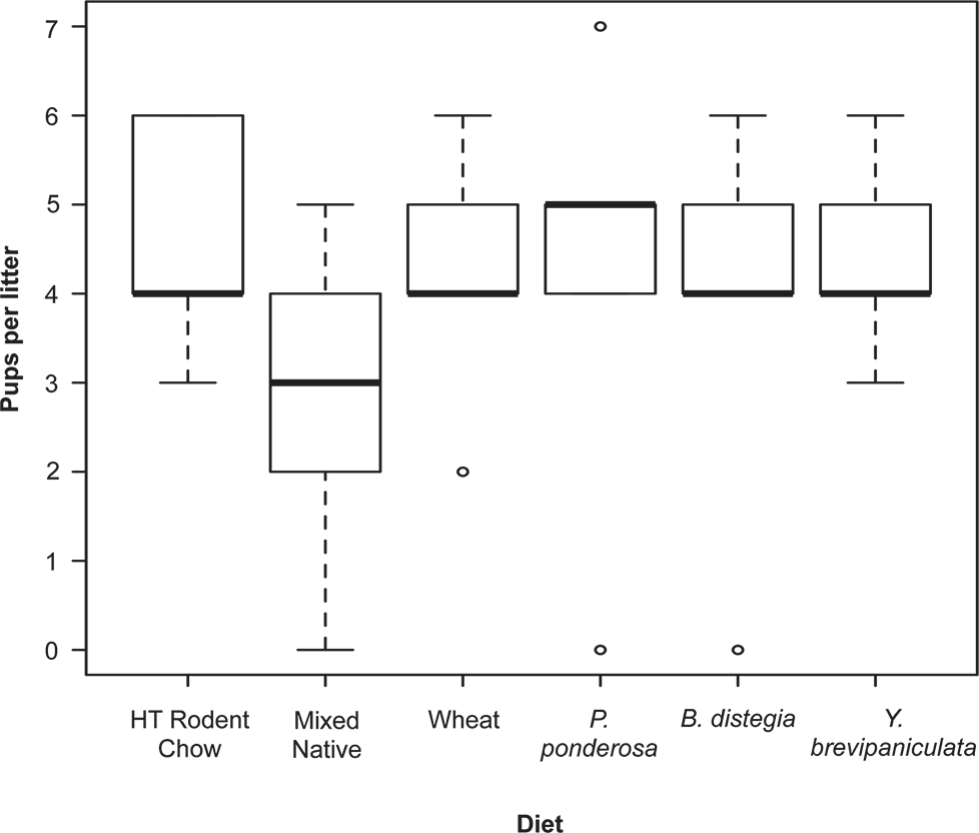
Box and whisker plot of number of pups per litter (at weaning) for each single-choice diet (open circles represent outliers which were included in the analysis). Although the median litter size (thickened bar) differs slightly, all treatments yielded a mean of four pups per litter, thereby producing an exponential growth rate assuming equal sex ratios among treatments.

Population growth of *P*. *maniculatus* can be markedly responsive to a rapidly available food: females enter estrus every five days, and pups are weaned and reproductive six weeks after conception [64]. Single choice diets had no significant effect on the rate of conception except for females fed a diet of pine seeds; these animals had a significantly lower rate of conception (Cochran’s Q, McNemar’s Test, P = 0.045). Neither the number of pups per litter (KW χ^2^ = 5.42, df = 5, P = 0.36, Fig 1) nor weight per pup at weaning (F_5, 60 = 1.553_, P = 0.187, mean: 17.33g/pup, see Supplemental materials) was significantly different among treatments.

**Fig 2:**
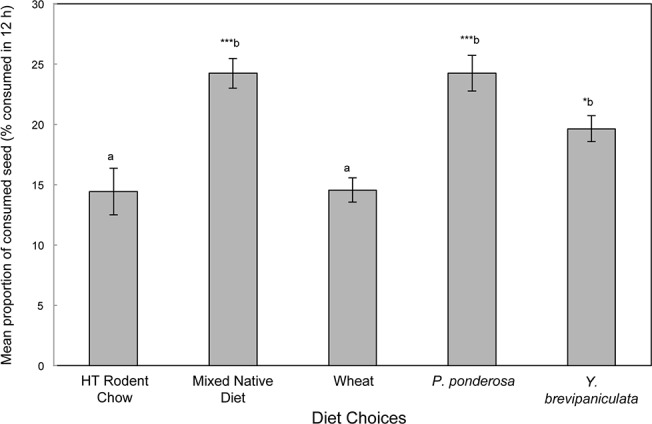
Seed choice based on proportion of the feed consumed in multiple-choice feeding trials. Deer mice consumed approximately 20% of their diet as bamboo (*Yushania brevipaniculata*) seeds (per 12 h); they consumed significantly more bamboo (P<0.05*) mixed native seeds (P<0.001***)(1:1 mix of *Balsamorhiza sagittata* and *Pseudoregnaria spicata*) and *Pinus ponderosa* seeds (P<0.001***) than locally grown wheat seeds or Harlan-Teklad Rodent Chow (ANOVA, F_3,8 = 3.45_, P < 0.0001).

In multiple-choice cafeteria-style feeding trials, we found that mice did not consume significantly more native seeds or pine seeds by mass than bamboo seeds (F = 2.07, df = 4, P > 0.09, Fig 2), and they preferred *P*. *ponderosa* seeds and the native seed mix to rodent laboratory feed and wheat seeds (F = 12.1, df = 4, P < 0.0001). Mice consumed approximately 4 g of feed per 12 h feeding period and consumed 20% of their diet as bamboo seeds (ranging from 18% to 26%, Fig 2). Seeds of *B*. *distegia*, *Y*. *brevipaniculata*, wheat, *P*. *spicata* (native grass), and Harlan Teklad Rodent Chow have approximately the same caloric content and are composed primarily of carbohydrates. Seeds of *P*. *ponderosa* and *B*. *sagittata*, in contrast, have a substantially higher caloric content, reflecting these seeds’ higher fat and protein content (S3 Table).

**Fig 3:**
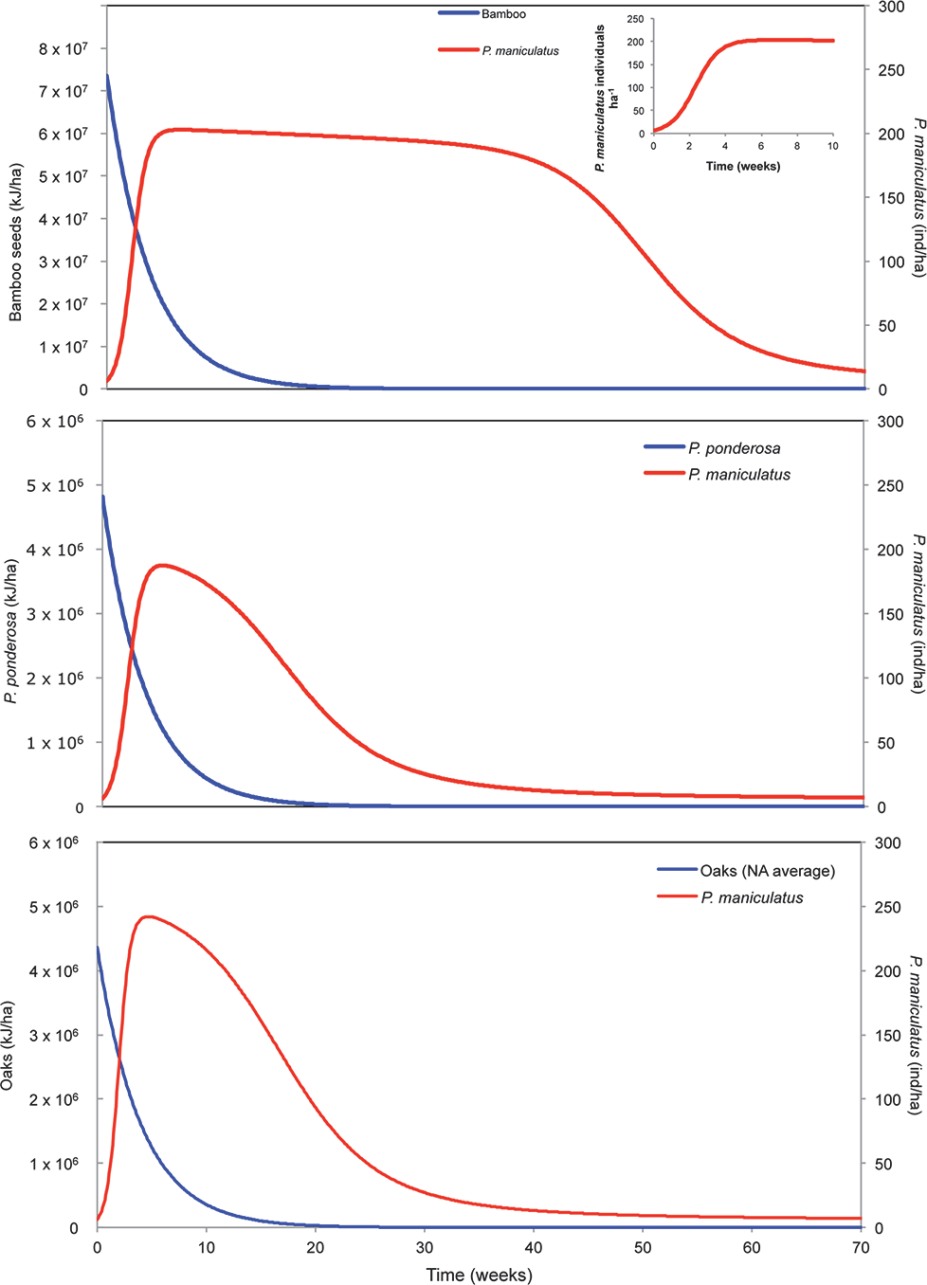
Joint dynamics of seeds ([a] bamboo, [b] oak and [c] pine) and deer mice densities following a mast event. Deer mice densities (dashed line) rise sharply in response to newly available seeds (solid line) to a new plateau several times higher than *M*_0_, their initial abundance. As mice consume seeds, they are initially only limited by their maximum rate of consumption (i.e. handling time [*h*]), not by supply, producing a prolonged quasi-equilibrium (QE). QE lasts until seed density falls below the immigration threshold and then again when deer mice become seed-limited again; seeds are depleted completely and the deer mice population returns to its pre-mast density. The QE period for bamboo lasts much longer than the other two native species and reaches a higher peak value than either oaks or pines. Parameter values for this figure are listed in Table 1 with derivations in S2 Table.

We incorporated estimates of the density and duration of bamboo seed production in Asia [52–54] along with reported *P*. *maniculatus* densities [56,57] into our modified Rosenzweig—MacArthur consumer-resource model (Eq 1–4) to assess the potential impact of bamboo mast fruiting on deer mice population growth (Fig 3). We also modeled *Pinus* and *Quercus* masting events in North America, to compare these previously measured events with our theoretical bamboo masting event. With the reproduction values we measured, the model predicts a time frame, based on mast length, during which mouse densities attain significantly higher levels (~150–200 mice per hectare) than normal observed mouse densities in these areas (8–20 mice per hectare) [65,66]. The time frame of the bamboo mast event is much longer than masting events among native pines and oaks. Consequently, mice could reach a higher maximum population density that can be sustained for longer due to the larger energy contribution from bamboo compared to native resources.

Dynamics of the mast consumer-resource system exhibit a sequence of distinct phases. In the first phase, the deer mice population increases exponentially at rate *r = β + ca-δ* until it reaches a “quasi-equilibrium” (QE), or maximum sustained density at population densities 5–7 fold greater than background levels. The initial exponential rate of increase, *r*, is derived by assuming that the initial seed supply is well above the attack rate half-saturation density (i.e., *B*_*0*_ *>> h*) and that intraspecific competition is extremely weak [viz., *M << K(B*_0_)]. Both this phase and the subsequent phase account for new immigrants arriving in the plot once *B* surpasses the energetic threshold to attract mice from adjacent areas.

Mice numbers during the subsequent quasi-equilibrium phase decline slowly and their abundance at any time is approximately a function of seed density alone. The QE densities of deer mice can be found by solving *dM*/*dt* = 0 for *M* assuming *B* >> *h*, which gives

$$M_{QE}(B)=K(B)(\beta +ca-\delta )$$ (Eq 5)

Mouse populations remain relatively stable until bamboo seeds fall below the immigration threshold, after which immigrants no longer arrive and the density of bamboo seeds *B* is approximately the same order as *h* (Fig 3). Once *B ~ h*, seed consumption is limited by supply rather than the consumption capacity of deer mice, and during this final phase, seed abundance falls precipitously and mice densities return to pre-mast levels, *M*_*0*_ (*B → 0*).

## Discussion

The population dynamics of the interaction are intrinsically driven by the amount and persistence of a new energy resource. Results of our feeding trials and modeling predictions for *P*. *maniculatus* are consistent with rapid population changes in rodent populations during bamboo masting events in Asia and South America [12,38,67]. We contend that a substantial risk of a similar sequence could arise in North America due to the rapid proliferation and expansion of non-native running bamboos within the range of *P*. *maniculatus*. As a result, mast fruiting by a naturalized bamboo species, or any potential naturalized masting species, could cause a rapid increase in *P*. *maniculatus* populations that persists for multiple generations as successive generations of deer mice feed on fallen bamboo seeds [26,27,68](Fig 3).

Extensive field sampling and subsequent simulations of deer mice populations indicate a similar influence of native species’ primary productivity on deer mice population dynamics [29,69]. A direct link between the incidence of infectious diseases and *Peromyscus* population irruptions is elusive, but a pronounced increase in the number of SNV cases in the Four Corners region of the U.S. in the 1990s nonetheless correlates to increases in seed production in the local vegetation, including masting by Piñon pines [70].

Our projection is based on relevant aspects of deer mice biology (e.g., life history characters, biotic interactions, population dynamics) within these communities and provides a realistic, if conservative, response scenario to a pulsed resource event. With a large addition of a novel food our model predicts a large numerical response in the *P*. *maniculatus* population, a result supported by field sampling [7,27,29,67]. Deer mice rapidly respond to changes in food availability by shifting diet proportions to access the most available resource [71]. As the population reaches a new numerical plateau provided by increased resources the population size has increased by several fold. This second phase is characterized by the addition of new members into the breeding pool, including immigrants. A food subsidy often extends the breeding season for currently reproductive *P*. *maniculatus* females and also provides resources for breeding to resume earlier in spring [27,28]. Many deer mice populations within the already naturalized range of bamboos (Oregon, Washington and California) breed year-round, and their response to bamboo masting could be even more pronounced than we modeled [72]. Additionally, the prolonged breeding season could enable young-of-the-year [73], the offspring born during the current reproductive season, to reproduce within their first season. This potential for reproduction in young-of-the-year females closely approximates exponential population growth in which all members of the population contribute equally [74]. The quasi-equilibrium phase persists because the plot of seed consumption by the mice reaches a plateau (expressed as *B >> h*, mice are limited by maximum consumption rate, not seed availability) and lasts until the bamboo seed density declines to the attack rate half-saturation density (*B ≈ h)*. In this phase the dynamics of predator satiation play out: mice are limited by a saturated seed density, leaving the excess seeds to germinate if unattacked by other seed predators [75,76]. Eventually (the third phase) the attack rate surpasses the seed abundance and intra-specific competition for food increases, and mouse density declines rapidly. Mouse density also declines rapidly due to diminishing food and correspondingly lowers population growth rates. Deer mice populations then reach the fourth and final phase in which they return to background pre-mast population densities. Results from our three modeled scenarios differ primarily in the second phase, the equilibrium. Bamboo is able to satiate seed predators for a much longer time than pines or oaks due to much higher output. Bamboos however, reproduce once during their lifecycle, whereas pines, oaks, and most other masting species may undergo several smaller mast events. The modeled event maintains the same shifts through the three phases, making it applicable to other consumer—resource events (e.g. *Pinus*, *Picea*, *Nothofagus*, *Fagus* and *Quercus* species (see[75]) with a single pulse in new resources [28,77](Fig 3B–3C).

Our model assumes that food availability is the primary factor determining deer mice population levels. Predation could conceivably exert a strong constraint on population growth. Experiments with food additions during and after active reproductive periods have however produced contrasting results. For instance, the numerical response in small populations of mice resulted in their increased predation and decreased survivorship [78], but *P*. *maniculatus* populations in Canada increased when presented with more food resources [27]. Although *P*. *maniculatus* is preyed upon by multiple avian and mammalian predators (e.g., raptors, coyotes) in these communities, experimental removal of its predators reveal these “top-down” forces collectively exert little influence on mice abundance [79]. In contrast, deer mice undergo a rapid numerical response to the “bottom up” effect of a food subsidy [27,28], paralleling the results in our feeding trials and the model’s predictions. Pearson and Fletcher [80] showed that the now abundant larvae of introduced gall flies (*Urophora* spp.), biological control agents on *Centaurea maculosa*, provide a substantial portion of deer mice diet from late spring through winter. As a result, deer mice abundance can be two fold greater when this subsidy is available compared with the abundance in controls. Ominously, *P*. *maniculatus* that tested seropositive for SNV were three times more abundant when this food subsidy was available. Although deer mice density increased as a direct consequence of the food addition, the rate of transmission of SNV among these mice may have been enhanced by the food subsidy due to resulting increased intraspecific interactions [7]. This rate of population increase is attributable to a numerical response among the mice, given their 28-day reproductive cycle and their ability to migrate to a food subsidy [27,81].

Deer mice produce litters of similar size when fed an equal biomass of seeds of either bamboo or the native grass *P*. *spicata*. Why then are surges in the size of deer mice populations not produced by this native food in the Pacific Northwest? Leptomorphic bamboos and *P*. *spicata* differ radically in habit, phenology and seed production, which likely translate into radically different outcomes for deer mice under field conditions. The caespitose *P*. *spicata*, has a much lower adult density even in habitats in which it is dominant than the culm density common among clonally spreading leptomorphic bamboos (cf. [82,83]). Furthermore, total seed mass per unit area in mature communities and duration of seed production are strikingly lower for *P*. *spicata* than the corresponding values for bamboos. A bamboo seed rain typically lasts for 6–18 months and can produce as much as 2000 kg of seeds per hectare [53,54]. Given the average biomass of *P*. *spicata* seeds [60], we estimate seed production for a steppe community with maximum (95%) reported cover of mature *P*. *spicata* [84] would be < 5 kg^-1^ha^-1^y^-1^. *Pseudoroegnaria spicata* occurs in few coniferous forests in the PNW; its maximum reported canopy cover in a forest understory is 80% but is commonly much lower [85]. In neither forest nor steppe communities would seed production of *P*. *spicata* support population growth for deer mice on the scale we predict here for a masting bamboo. Although deer mice are capable of deriving the majority of their nutrients from seeds, these low-protein and low-fat foods must be supplemented by insects with high protein and fat contents [86]. Insect populations also experience numerical increases following a masting event [87], which could directly support growing rodent populations [76].

### *Peromyscus maniculatus*—A Persistent Threat to Forests and Crops

Upon depletion of the seed subsidy, starving omnivorous deer mice would disperse in search of food and rapidly encounter crops in much of the interior PNW. The region’s native steppe has been largely converted to the production of cereals (e.g., wheat, oats, barley) and legumes (lentils, dry peas, alfalfa) [88,89], which are readily consumed by deer mice [90]. For example, in eastern Washington and northern Idaho much farmland lies adjacent to low elevation *P*. *ponderosa* forest, native habitat for deer mice. Soil survey maps [88,89] delineate the long, interdigitating ecotone across which mice readily move between forest habitat and fields [90,66]. In steppe and forest habitats, seed consumption from deer mice alters seed recruitment, especially for species with high palatability (e.g. *P*. *ponderosa*, *Lupinus sericeus*, *Pinus contorta*, *Lithosperma ruderale*, among others) [91,92]. Even if an outbreak of deer mice were foreseen, controlling, much less eradicating, the immigration of mice into crop fields would likely prove challenging. A rodenticide, such as zinc phosphide, can be spread as pellets or impregnated on grain outside the fields but is only temporarily effective [90].

### Importation of Ornamental Bamboos: New Opportunities for Spread and Naturalization

Use of ornamental running bamboos in landscaping is increasing in the U.S., as indicated by the steady 30-year increase of the number of species available on the American Bamboo Society’s species list (http://www.bamboo.org/BambooSourceList). As the array of bamboo species in the horticulture trade increases, so does the likelihood that one or more of these species will escape cultivation and establish large, persistent populations. On the U.S. East Coast, large infestations of *Phyllostachys* spp. have spread from abandoned plantings into forest understories (http://www.eddmaps.org, RNM, pers. observation). In the PNW, escaped *P*. *japonica* is locally and repeatedly naturalized in coastal forests dominated by *Pseudotsuga menziesii* adjacent to old residences (http://www.eddmaps.org, MCS, unpublished data); these incursions are not surprising given the extraordinary shade tolerance among the temperate Asian bamboos [22].

As a result, coniferous forests along the North American West Coast and inland, in which *P*. *maniculatus* is common and locally abundant [93], may be particularly at risk of naturalization and even invasion by bamboos. These forests share strong floristic and environmental similarities with Asian coniferous forests in which *Sasa kurilensis* and other dwarf (< 2 m) leptomorphic bamboos, commonly form a continuous hedge-like layer [52,94]. Extensive naturalization of leptomorphic bamboos with aggressively spreading rhizomes could eventually produce massive seed crops in PNW forests. In a worst-case scenario a naturalized bamboo population undergoes mass flowering/fruiting on forested public lands; resident *P*. *maniculatus* populations rapidly increase, the increase driven by the newly available resource, followed by the bamboo soon waning as a food source. As a consequence, starving peridomestic *P*. *maniculatus* seek food in structures, including dwellings [95] with subsequent public health implications.

### Preemptively Curbing a Bamboo-Deer mouse Link

Results of our experiments and model analyses meet the premise of the precautionary principle [96], i.e., justifying measures to be taken before a larger body of retrospective evidence has been assembled on a link between masting in leptomorphic bamboos and the population dynamics of deer mice in coniferous forests. In effect, our conclusions are an early warning from which deliberate action can be taken. Two revisions to U.S. and Canadian plant quarantine policy [97,98] regarding the importation and distribution of bamboos now seem advisable. Early Detection/Rapid Response (ED/RR), i.e., deliberate steps now taken to detect and eradicate aggressively spreading non-native plants, as practiced in U.S. National Parks (http://www.nature.nps.gov/biology/invasivespecies/EPMT_teams.cfm), needs to be directed at non-native bamboos on public lands. Second, any leptomorphic bamboo’s flowering interval and the palatability of its seeds for rodents (and other seed predators) in its native range should be included in evaluating its potential introduction into the U.S. This information could then be coupled with results from experimental gardens in a new range that gauge the species’ potential to spread vegetatively and persist [99].

## Supporting Information

S1 Dataset Single-choice feeding trial. Raw, untransformed data from single-choice feeding trials to determine survivability of single choice diets.
(PDF)

S2 Dataset Single-choice reproductive feeding trial. Raw, untransformed data from combined naïve and experienced females for reproductive output, single-choice feeding trial.
(PDF)

S3 Dataset Multiple-choice feeding trial. Raw, untransformed data from multiple-choice feeding trials done with 10 individual mice.
(PDF)

S1 Table Bamboo flowering and fruiting records from National Herbarium (US). All flowering events took place in cultivated patches in the USA. All flowering bamboos formed caryopses with rates from 40–100% in the introduced range including naturalized species, *Phyllostachys aurea* and *Sasa palmata*.
(DOCX)

S2 Table Supporting Information. Calculations, derivations and r-code for the maximum seed attack rate (*a*) and the seed density at attack rate half saturation (*h*).
(DOCX)

S3 Table Caloric content of seeds used in our study. All measurements are an average (± SE) of three 5-g samples that were ground and combusted in an oxygen bomb calorimeter.
(DOCX)
