# Correction: Potential Role of Masting by Introduced Bamboos in Deer Mice (*Peromyscus maniculatus*) Population Irruptions Holds Public Health Consequences

**DOI:** 10.1371/journal.pone.0129414

**Published:** 2015-06-03

**Authors:** Melissa C. Smith, Richard Gomulkiewicz, Richard N. Mack

There is an error in Eq 4 in the Materials and Methods section. Please view the complete, correct equation here:

$$M_{0}=\;(\beta -\delta )*K_{min}$$ (Eq 4)
