# Supplementary material for: Potential Role of Masting by Introduced Bamboos in Deer Mice (Peromyscus maniculatus) Population Irruptions Holds Public Health Consequences
Source: PLoS One. 2015 Apr 21;10(4):e0124419. doi: 10.1371/journal.pone.0124419 (PMC4405191; doi:10.1371/journal.pone.0124419)
Supplement: S1 Dataset — Raw, untransformed data from single-choice feeding trials to determine survivability of single choice diets. (PDF) [file pone.0124419.s001.pdf]

date	cage	shelf	treatment	MW1	MW2	tot.wt
24-Dec-08	1	1	control	22	23.9	45.9
24-Dec-08	3	1	pine	17.7	17.7	35.4
24-Dec-08	4	1	grass	18.5	17.7	36.2
24-Dec-08	4	2	pine	17.5	16.5	34
24-Dec-08	3	2	grass	17.35	17.35	34.7
24-Dec-08	2	2	control	18.9	17.4	36.3
24-Dec-08	1	2	bamboo	21.2	17.8	39
24-Dec-08	1	3	control	20.6	18.9	39.5
24-Dec-08	2	3	bamboo	20	19.1	39.1
24-Dec-08	3	3	pine	19.6	25.6	45.2
24-Dec-08	4	3	grass	18.8	20.2	39
24-Dec-08	1	4	bamboo	15.6	18.1	33.7
24-Dec-08	2	4	pine	21.4	19.3	40.7
24-Dec-08	3	4	control	18.9	18.1	37
24-Dec-08	4	4	grass	18.5	19.7	38.2
26-Dec-08	1	1	control	24.1	21.4	45.5
26-Dec-08	2	1	bamboo	17.6	18.2	35.8
26-Dec-08	3	1	pine	16.6	16.6	33.2
26-Dec-08	4	1	grass	17.3	18.2	35.5
26-Dec-08	1	2	bamboo	17.4	20.2	37.6
26-Dec-08	2	2	control	18.5	16.9	35.4
26-Dec-08	3	2	grass	16.8	16.4	33.2
26-Dec-08	4	2	pine	17.1	16.3	33.4
26-Dec-08	1	3	control	22	19.3	41.3
26-Dec-08	2	3	bamboo	20.4	19.2	39.6
26-Dec-08	3	3	pine	18.2	23.6	41.8
26-Dec-08	4	3	grass	17.3	19.2	36.5
26-Dec-08	4	4	grass	18.6	17.2	35.8
26-Dec-08	1	4	bamboo	15.6	18	33.6
26-Dec-08	2	4	pine	19	23	42
26-Dec-08	3	4	control	19.5	19.5	39
1-Jan-09	4	2	pine	15.7	16.6	32.3
1-Jan-09	1	2	bamboo	16.9	20.4	37.3
1-Jan-09	2	2	control	19.1	17.4	36.5
1-Jan-09	3	2	grass	17.1	16.1	33.2
1-Jan-09	1	1	control	24.3	21.5	45.8
1-Jan-09	3	1	pine	17	17	34
1-Jan-09	4	1	grass	18.6	17.8	36.4
1-Jan-09	1	3	control	20	27.1	47.1
1-Jan-09	2	3	bamboo	18.8	18.9	37.7
1-Jan-09	3	3	pine	18	17.5	35.5
1-Jan-09	4	3	grass	17.4	19.8	37.2

1-Jan-09	4	4	grass	17.6	18.9	36.5
1-Jan-09	3	4	control	19.6	19.8	39.4
1-Jan-09	2	4	pine	19.3	20	39.3
1-Jan-09	1	4	bamboo	17.3	15.4	32.7
2-Jan-09	1	1	control	24.8	21.9	46.7
2-Jan-09	2	1	pine	17.8	17.8	35.6
2-Jan-09	4	1	grass	17.6	18.4	36
2-Jan-09	1	2	bamboo	17.2	20.5	37.7
2-Jan-09	2	2	control	17.2	18.7	35.9
2-Jan-09	3	2	grass	17	15.9	32.9
2-Jan-09	4	2	pine	17.6	17.1	34.7
2-Jan-09	1	3	control	21.8	20.1	41.9
2-Jan-09	2	3	bamboo	19.3	20.1	39.4
2-Jan-09	3	3	pine	18.5	20.4	38.9
2-Jan-09	4	3	grass	17.5	19.6	37.1
2-Jan-09	1	4	bamboo	15.5	17.2	32.7
2-Jan-09	2	4	pibe	19.4	20.3	39.7
2-Jan-09	3	4	control	19.6	19.4	39
2-Jan-09	4	4	grass	19.1	17.5	36.6
5-Jan-09	1	1	control	22.8	25.3	48.1
5-Jan-09	3	1	Pine	18.7	18.7	37.4
5-Jan-09	4	1	grass	17.7	18.7	36.4
5-Jan-09	1	2	bamboo	17.5	20.7	38.2
5-Jan-09	2	2	control	18.8	17.4	36.2
5-Jan-09	3	2	grass	16.1	17.3	33.4
5-Jan-09	4	2	pine	17.4	17.3	34.7
5-Jan-09	1	3	control	21.5	19.6	41.1
5-Jan-09	2	3	bamboo	19.9	19	38.9
5-Jan-09	3	3	pine	18.8	21.3	40.1
5-Jan-09	4	3	grass	172	20.2	192.2
5-Jan-09	1	4	bamboo	17.2	15.5	32.7
5-Jan-09	2	4	pine	20.9	21.7	42.6
5-Jan-09	3	4	control	19.1	19.4	38.5
5-Jan-09	4	4	grass	20.1	17.8	37.9
7-Jan-09	1	1	control	25	22.4	47.4
7-Jan-09	3	1	pine	19.3	19.3	38.6
7-Jan-09	4	1	grass	17.2	17.9	35.1
7-Jan-09	1	2	bamboo	17.9	20.8	38.7
7-Jan-09	2	2	control	18.6	17.1	35.7
7-Jan-09	3	2	grass	15.9	17.4	33.3
7-Jan-09	4	2	pine	17.2	17.2	34.4
7-Jan-09	1	3	control	21.3	19.3	40.6
7-Jan-09	2	3	bamboo	19.9	19.5	39.4

7-Jan-09	3	3 pine	18.9	21.9	40.8
7-Jan-09	4	3 grass	17.4	19.7	37.1
7-Jan-09	1	4 bamboo	15.6	16.9	32.5
7-Jan-09	2	4 pine	22	21.6	43.6
7-Jan-09	3	4 control	19.5	19.4	38.9
7-Jan-09	4	4 grass	19.7	17.3	37
8-Jan-09	1	1 mean	22.5	24.3	46.8
8-Jan-09	3	1 pine	19.2	19.2	38.4
8-Jan-09	4	1 grass	17.8	17.4	35.2
8-Jan-09	1	2 bamboo	17.7	20.6	38.3
8-Jan-09	2	2 control	18.6	16.6	35.2
8-Jan-09	3	2 grass	17.4	16.2	33.6
8-Jan-09	4	2 pine	17.8	17.7	35.5
8-Jan-09	1	3 control	19.5	20.9	40.4
8-Jan-09	2	3 bamboo	19.7	19.3	39
8-Jan-09	3	3 pine	22.6	18.9	41.5
8-Jan-09	4	3 grass	17.4	19.6	37
8-Jan-09	1	4 grass	16.9	19.7	36.6
8-Jan-09	2	4 control	20.2	18.7	38.9
8-Jan-09	3	4 pine	22.1	21.2	43.3
8-Jan-09	4	4 bamboo	15.2	16.9	32.1
9-Jan-09	1	1 mean	24.6	22.5	47.1
9-Jan-09	3	1 pine	18.8	18.8	37.6
9-Jan-09	4	1 grass	17.1	17.5	34.6
9-Jan-09	1	2 bamboo	17.4	20.5	37.9
9-Jan-09	2	2 control	18.5	16.7	35.2
9-Jan-09	3	2 grass	15.8	17.2	33
9-Jan-09	4	2 pine	17.5	17.5	35
9-Jan-09	1	3 control	19.8	20.7	40.5
9-Jan-09	2	3 bamboo	19.6	19.2	38.8
9-Jan-09	3	3 pine	23.1	19.2	42.3
9-Jan-09	4	3 grass	17.1	19.6	36.7
9-Jan-09	1	4 bamboo	15	16.6	31.6
9-Jan-09	2	4 pine	21.3	22.4	43.7
9-Jan-09	3	4 control	19.7	19.3	39
9-Jan-09	4	4 grass	20.3	17.3	37.6
11-Jan-09	1	1 mean	22.1	24.7	46.8
11-Jan-09	3	1 pine	18.9	18.9	37.8
11-Jan-09	4	1 grass	17.4	17.5	34.9
11-Jan-09	1	2 bamboo	18	17.9	35.9
11-Jan-09	2	2 control	16	16.8	32.8
11-Jan-09	3	2 grass	18.6	16.7	35.3
11-Jan-09	4	2 pine	21.1	17.6	38.7

11-Jan-09	1	3	control	21.9	20.4	42.3
11-Jan-09	2	3	bamboo	19.6	18.9	38.5
11-Jan-09	3	3	pine	24.4	18.8	43.2
11-Jan-09	4	3	grass	17.3	20	37.3
11-Jan-09	1	4	bamboo	16.6	14.9	31.5
11-Jan-09	2	4	pine	21.5	22.9	44.4
11-Jan-09	3	4	control	18.6	19.5	38.1
11-Jan-09	4	4	grass	20.5	17.6	38.1
13-Jan-09	1	1	mean	22.2	24.4	46.6
13-Jan-09	3	1	pine	18.9	18.9	37.8
13-Jan-09	4	1	grass	17.2	17.3	34.5
13-Jan-09	1	2	bamboo	21.6	18	39.6
13-Jan-09	2	2	control	19.1	17.4	36.5
13-Jan-09	3	2	grass	16.2	17	33.2
13-Jan-09	4	2	pine	17.4	17.5	34.9
13-Jan-09	1	3	control	21.9	21.3	43.2
13-Jan-09	2	3	bamboo	19.6	19.3	38.9
13-Jan-09	3	3	pine	25	18.6	43.6
13-Jan-09	4	3	grass	17.6	20.4	38
13-Jan-09	1	4	bamboo	17.2	15.6	32.8
13-Jan-09	2	4	pine	22.1	22.2	44.3
13-Jan-09	3	4	control	19	19.8	38.8
13-Jan-09	4	4	grass	21.1	17.8	38.9
15-Jan-09	1	1	mean	22	22.9	44.9
15-Jan-09	3	1	pine	18.7	18.7	37.4
15-Jan-09	4	1	grass	17.6	17.4	35
15-Jan-09	1	2	bamboo	17.8	21.5	39.3
15-Jan-09	2	2	control	18.7	16.9	35.6
15-Jan-09	3	2	grass	16.2	17.1	33.3
15-Jan-09	4	2	pine	17.8	18.2	36
15-Jan-09	1	3	control	21.4	21.1	42.5
15-Jan-09	2	3	bamboo	18.9	19.4	38.3
15-Jan-09	3	3	pine	25.9	18.7	44.6
15-Jan-09	4	3	grass	20	17.1	37.1
15-Jan-09	1	4	bamboo	17.1	15.5	32.6
15-Jan-09	2	4	pine	22.2	21.7	43.9
15-Jan-09	3	4	control	19.2	19.9	39.1
15-Jan-09	4	4	grass	20.9	17.4	38.3
17-Jan-09	1	1	mean	25	21.3	46.3
17-Jan-09	3	1	pine	18.6	18.6	37.2
17-Jan-09	4	1	grass	16.7	17.1	33.8
17-Jan-09	1	2	bamboo	17.6	20.9	38.5
17-Jan-09	2	2	control	17.1	18.1	35.2

17-Jan-09	3	2 grass	15.7	16.5	32.2
17-Jan-09	4	2 pine	17.2	16.6	33.8
17-Jan-09	1	3 control	21	21.4	42.4
17-Jan-09	2	3 bamboo	19.3	18.7	38
17-Jan-09	3	3 pine	18.8	26.3	45.1
17-Jan-09	4	3 grass	17.2	19.7	36.9
17-Jan-09	1	4 bamboo	17.1	15.1	32.2
17-Jan-09	2	4 pine	21.9	22.8	44.7
17-Jan-09	3	4 control	19.8	18.4	38.2
17-Jan-09	4	4 grass	17.2	20.5	37.7
18-Jan-09	1	1 mean	21.1	23.2	44.3
18-Jan-09	3	1 pine	18.6	18.6	37.2
18-Jan-09	4	1 grass	17	17.4	34.4
18-Jan-09	1	2 bamboo	21	17.3	38.3
18-Jan-09	2	2 control	17.3	18.4	35.7
18-Jan-09	3	2 grass	16.3	17.2	33.5
18-Jan-09	4	2 pine	17.3	17.1	34.4
18-Jan-09	1	3 control	21.7	21.2	42.9
18-Jan-09	2	3 bamboo	19.2	19	38.2
18-Jan-09	3	3 pine	26.6	18.6	45.2
18-Jan-09	4	3 grass	20.2	17	37.2
18-Jan-09	1	4 bamboo	15.1	26.8	41.9
18-Jan-09	2	4 pine	21.7	22.7	44.4
18-Jan-09	3	4 control	19.4	19.2	38.6
18-Jan-09	4	4 grass	21.2	17.6	38.8
