# Supplementary material for: Potential Role of Masting by Introduced Bamboos in Deer Mice (Peromyscus maniculatus) Population Irruptions Holds Public Health Consequences
Source: PLoS One. 2015 Apr 21;10(4):e0124419. doi: 10.1371/journal.pone.0124419 (PMC4405191; doi:10.1371/journal.pone.0124419)
Supplement: S2 Dataset — Raw, untransformed data from combined naïve and experienced females for reproductive output, single-choice feeding trial. (PDF) [file pone.0124419.s002.pdf]

Feed	Pups
Harlan Teklad Rodent Chow	6
Harlan Teklad Rodent Chow	6
Harlan Teklad Rodent Chow	4
Harlan Teklad Rodent Chow	3
Harlan Teklad Rodent Chow	4
Harlan Teklad Rodent Chow	3
Harlan Teklad Rodent Chow	5
Harlan Teklad Rodent Chow	0
Harlan Teklad Rodent Chow	4
Harlan Teklad Rodent Chow	0
Mixed native	3
Mixed native	2
Mixed native	0
Mixed native	5
Mixed native	4
Mixed native	3
Mixed native	4
Mixed native	4
Mixed native	4
Mixed native	3
Wheat	4
Wheat	2
Wheat	4
Wheat	5
Wheat	6
Wheat	4
Wheat	4
Wheat	2
Wheat	5
Wheat	4
Pine	7
Pine	0
Pine	5
Pine	4
Pine	0
Pine	0
Pine	0
Pine	0
Pine	5
Pine	0
Pine	4
Pine	4

```
> aov.feed = aov(Pups~Feed)
Error in eval(expr, envir, enclos) : object
> attach(feed)
> aov.feed = aov (Pups ~ Feed)
> aov.feed
Call:
   aov(formula = Pups ~ Feed)

Terms:
                Feed Residuals
Sum of Squares   25.25606 195.18333
Deg. of Freedom        5        60

Residual standard error: 1.803623
Estimated effects may be unbalanced
> summary(aov.feed)
            Df Sum Sq Mean Sq F value Pr(>F
Feed         5  25.26   5.051   1.553  0.187
Residuals   60 195.18   3.253
>
```

B. distegia	0
B. distegia	4
B. distegia	5
B. distegia	6
B. distegia	4
B. distegia	5
B. distegia	4
B. distegia	5
B. distegia	5
B. distegia	4
B. distegia	2
B. distegia	6
Y. brevipaniculata	4
Y. brevipaniculata	4
Y. brevipaniculata	3
Y. brevipaniculata	5
Y. brevipaniculata	6
Y. brevipaniculata	3
Y. brevipaniculata	4
Y. brevipaniculata	3
Y. brevipaniculata	5
Y. brevipaniculata	3

: 'Pups' not found

)
