# Supplementary material for: Potential Role of Masting by Introduced Bamboos in Deer Mice (Peromyscus maniculatus) Population Irruptions Holds Public Health Consequences
Source: PLoS One. 2015 Apr 21;10(4):e0124419. doi: 10.1371/journal.pone.0124419 (PMC4405191; doi:10.1371/journal.pone.0124419)
Supplement: S3 Dataset — Raw, untransformed data from multiple-choice feeding trials done with 10 individual mice. (PDF) [file pone.0124419.s003.pdf]

Trt	Mouse	Wk	Remaining	Consumed	Rel abun	average
Bamboo	1	1	2.6	0.4	18.1818182	19.5123876
Bamboo	1	2	2.4	0.6	12.1212121	
Bamboo	1	3	1.8	1.2	25.5319149	
Bamboo	1	4	1.6	1.4	30.4347826	
Bamboo	1	5	2.3	0.7	24.137931	
Bamboo	1	6	2.8	0.2	6.66666667	
Control	1	1	2.5	0.5	22.7272727	12.9616833
Control	1	2	2.7	0.3	9.09090909	
Control	1	3	2.6	0.4	8.5106383	
Control	1	4	2.7	0.3	6.52173913	
Control	1	5	2.2	0.8	27.5862069	
Control	1	6	2.9	0.1	3.33333333	
Mixed	1	1	2	1	22.7272727	23.4028666
Mixed	1	2	2.5	0.5	15.1515152	
Mixed	1	3	2.8	0.2	25.5319149	
Mixed	1	4	1.8	1.2	26.0869565	
Mixed	1	5	2.2	0.8	27.5862069	
Mixed	1	6	2.3	0.7	23.3333333	
Pine	1	1	2.4	0.6	27.2727273	23.6013312
Pine	1	2	1.1	1.9	27.2727273	
Pine	1	3	1.3	1.7	14.893617	
Pine	1	4	1.7	1.3	28.2608696	
Pine	1	5	2.5	0.5	17.2413793	
Pine	1	6	2.2	0.8	26.6666667	
Wheat	1	1	2.8	0.2	9.09090909	14.4106203
Wheat	1	2	1.8	1.2	36.3636364	
Wheat	1	3	1.8	1.2	25.5319149	
Wheat	1	4	2.6	0.4	8.69565217	
Wheat	1	5	2.9	0.1	3.44827586	
Wheat	1	6	2.9	0.1	3.33333333	
Bamboo	2	1	2.9	0.1	25.7142857	22.1840117
Bamboo	2	2	2.1	0.9	17.6470588	
Bamboo	2	3	1.8	1.2	27.9069767	
Bamboo	2	4	2.3	0.7	19.4444444	
Bamboo	2	5	2.8	0.2	25	
Bamboo	2	6	2.6	0.4	17.3913044	
Control	2	1	2.9	0.1	11.4285714	22.9298664
Control	2	2	1.9	1.1	21.5686275	
Control	2	3	1.9	1.1	25.5813954	
Control	2	4	1.1	1.9	52.7777778	
Control	2	5	2.5	0.5	21.875	
Control	2	6	2.9	0.1	4.34782609	

Mixed	2	1	2.7	0.3	8.57142857	23.9423209
Mixed	2	2	1.6	1.4	27.4509804	
Mixed	2	3	1.9	1.1	25.5813954	
Mixed	2	4	2.8	0.2	5.55555556	
Mixed	2	5	2.5	0.5	15.625	
Mixed	2	6	1.6	1.4	60.8695652	
Pine	2	1	2.9	0.1	31.4285714	24.5246413
Pine	2	2	2.2	0.8	15.6862745	
Pine	2	3	2.6	0.4	9.30232558	
Pine	2	4	2.6	0.4	11.1111111	
Pine	2	5	2.8	0.2	18.75	
Pine	2	6	1.6	1.4	60.8695652	
Wheat	2	1	2.9	0.1	22.8571429	15.114812
Wheat	2	2	1.9	1.1	17.6470588	
Wheat	2	3	2.5	0.5	11.627907	
Wheat	2	4	2.6	0.4	11.1111111	
Wheat	2	5	2.9	0.1	18.75	
Wheat	2	6	2.8	0.2	8.69565217	
Bamboo	3	1	2.6	0.4	14.2857143	15.1047435
Bamboo	3	2	2.7	0.3	6.66666667	
Bamboo	3	3	2.2	0.8	18.6046512	
Bamboo	3	4	2.9	0.1	2.85714286	
Bamboo	3	5	1.7	1.3	23.2142857	
Bamboo	3	6	2.5	0.5	25	
Control	3	1	2.4	0.6	21.4285714	14.2368033
Control	3	2	1.8	1.2	26.6666667	
Control	3	3	2.9	0.1	2.3255814	
Control	3	4	2.5	0.5	14.2857143	
Control	3	5	2.4	0.6	10.7142857	
Control	3	6	2.8	0.2	10	
Mixed	3	1	2.4	0.6	21.4285714	28.749231
Mixed	3	2	1.3	1.7	37.7777778	
Mixed	3	3	1.9	1.1	41.8604651	
Mixed	3	4	2.3	0.7	20	
Mixed	3	5	1.8	1.2	21.4285714	
Mixed	3	6	2.4	0.6	30	
Pine	3	1	2.1	0.9	32.1428571	32.9686539
Pine	3	2	2	1	22.2222222	
Pine	3	3	1.7	1.3	30.2325581	
Pine	3	4	0.9	2.1	60	
Pine	3	5	1.7	1.3	23.2142857	
Pine	3	6	2.4	0.6	30	
Wheat	3	1	2.7	0.3	10.7142857	8.94056848

Wheat	3	2	2.7	0.3	6.66666667	
Wheat	3	3	2.7	0.3	6.97674419	
Wheat	3	4	2.9	0.1	2.85714286	
Wheat	3	5	1.8	1.2	21.4285714	
Wheat	3	6	2.9	0.1	5	
Bamboo	4	1	2.5	0.5	15.625	24.2671779
Bamboo	4	2	2.7	0.3	28.2608696	
Bamboo	4	3	2.1	0.9	20.9302326	
Bamboo	4	4	2	1	18.8679245	
Bamboo	4	5	2.5	0.5	32.6086957	
Bamboo	4	6	1.3	1.7	29.3103448	
Control	4	1	2.8	0.2	6.25	4.85737619
Control	4	2	2.9	0.1	2.17391304	
Control	4	3	2.7	0.3	6.97674419	
Control	4	4	2.8	0.2	3.77358491	
Control	4	5	2.7	0.3	6.52173913	
Control	4	6	2.8	0.2	3.44827586	
Mixed	4	1	2.3	0.7	21.875	24.6365392
Mixed	4	2	0.5	2.5	32.6086957	
Mixed	4	3	2.1	0.9	20.9302326	
Mixed	4	4	1.3	1.7	32.0754717	
Mixed	4	5	1	2	28.2608696	
Mixed	4	6	2.3	0.7	12.0689655	
Pine	4	1	2.1	0.9	28.125	22.2725809
Pine	4	2	1.1	1.9	19.5652174	
Pine	4	3	1.7	1.3	30.2325581	
Pine	4	4	1.1	1.9	24.5283019	
Pine	4	5	1.1	1.9	17.3913044	
Pine	4	6	2.2	0.8	13.7931035	
Wheat	4	1	2.1	0.9	28.125	19.0812685
Wheat	4	2	1.2	1.8	17.3913044	
Wheat	4	3	2.1	0.9	20.9302326	
Wheat	4	4	1.9	1.1	20.754717	
Wheat	4	5	1.7	1.3	15.2173913	
Wheat	4	6	2.3	0.7	12.0689655	
Bamboo	5	1	2.6	0.4	32.1428571	19.5290086
Bamboo	5	2	2.5	0.5	14.7058824	
Bamboo	5	3	2.8	0.2	6.06060606	
Bamboo	5	4	2.6	0.4	11.7647059	
Bamboo	5	5	2.8	0.2	27.5	
Bamboo	5	6	2.7	0.3	25	
Control	5	1	2.3	0.7	25	20.5852644
Control	5	2	2.3	0.7	20.5882353	

Control	5	3	2.8	0.2	6.06060606	
Control	5	4	2.2	0.8	23.5294118	
Control	5	5	2.4	0.6	15	
Control	5	6	2.6	0.4	33.3333333	
Mixed	5	1	2.5	0.5	17.8571429	29.8500764
Mixed	5	2	1.7	1.3	38.2352941	
Mixed	5	3	2.1	0.9	27.2727273	
Mixed	5	4	1.7	1.3	38.2352941	
Mixed	5	5	1.3	1.7	32.5	
Mixed	5	6	2.7	0.3	25	
Pine	5	1	2.7	0.3	17.8571429	28.8139801
Pine	5	2	2.2	0.8	23.5294118	
Pine	5	3	1.1	1.9	57.5757576	
Pine	5	4	2.3	0.7	20.5882353	
Pine	5	5	1.8	1.2	20	
Pine	5	6	2.6	0.4	33.3333333	
Wheat	5	1	2.9	0.1	7.14285714	12.3327816
Wheat	5	2	2.9	0.1	2.94117647	
Wheat	5	3	2.9	0.1	3.03030303	
Wheat	5	4	2.8	0.2	5.88235294	
Wheat	5	5	2.8	0.2	5	
Wheat	5	6	2.4	0.6	50	
Bamboo	6	1	1.9	1.1	36.6666667	18.9386682
Bamboo	6	2	2.6	0.4	29.787234	
Bamboo	6	3	2.6	0.4	8.88888889	
Bamboo	6	4	2.5	0.5	9.61538462	
Bamboo	6	5	2.2	0.8	22.2222222	
Bamboo	6	6	2.8	0.2	6.4516129	
Control	6	1	2.4	0.6	20	9.20269472
Control	6	2	2.8	0.2	4.25531915	
Control	6	3	2.3	0.7	15.5555556	
Control	6	4	2.8	0.2	3.84615385	
Control	6	5	2.7	0.3	8.33333333	
Control	6	6	2.9	0.1	3.22580645	
Mixed	6	1	2.7	0.3	10	21.3853643
Mixed	6	2	0.4	2.6	29.787234	
Mixed	6	3	1.2	1.8	40	
Mixed	6	4	1.8	1.2	23.0769231	
Mixed	6	5	2.2	0.8	22.2222222	
Mixed	6	6	2.9	0.1	3.22580645	
Pine	6	1	2.3	0.7	23.3333333	25.8701533
Pine	6	2	1.7	1.3	27.6595745	
Pine	6	3	1.9	1.1	24.4444444	

Pine	6	4	1.4	1.6	30.7692308	
Pine	6	5	1.7	1.3	36.1111111	
Pine	6	6	2.6	0.4	12.9032258	
Wheat	6	1	2.7	0.3	10	12.7751624
Wheat	6	2	2.6	0.4	8.5106383	
Wheat	6	3	2.5	0.5	11.1111111	
Wheat	6	4	1.3	1.7	32.6923077	
Wheat	6	5	2.6	0.4	11.1111111	
Wheat	6	6	2.9	0.1	3.22580645	
Bamboo	7	1	2.1	0.9	31.0344828	18.6433236
Bamboo	7	2	2.5	0.5	12.5	
Bamboo	7	3	2.7	0.3	10.3448276	
Bamboo	7	4	1.7	1.3	21.9512195	
Bamboo	7	5	2.7	0.3	12.5	
Bamboo	7	6	2.2	0.8	23.5294118	
Control	7	1	2.6	0.4	13.7931035	11.6654367
Control	7	2	2.3	0.7	17.5	
Control	7	3	2.8	0.2	6.89655172	
Control	7	4	2.3	0.7	12.195122	
Control	7	5	2.6	0.4	16.6666667	
Control	7	6	2.9	0.1	2.94117647	
Mixed	7	1	2.4	0.6	20.6896552	18.2571654
Mixed	7	2	2.6	0.4	10	
Mixed	7	3	1.6	1.4	48.2758621	
Mixed	7	4	2.2	0.8	12.195122	
Mixed	7	5	2.7	0.3	12.5	
Mixed	7	6	2.8	0.2	5.88235294	
Pine	7	1	2.6	0.4	13.7931035	25.3660117
Pine	7	2	1.7	1.3	32.5	
Pine	7	3	2.1	0.9	31.0344828	
Pine	7	4	1.7	1.3	26.8292683	
Pine	7	5	2.2	0.8	33.3333333	
Pine	7	6	2.5	0.5	14.7058824	
Wheat	7	1	2.4	0.6	20.6896552	17.7347293
Wheat	7	2	1.9	1.1	27.5	
Wheat	7	3	2.9	0.1	3.44827586	
Wheat	7	4	1.5	1.5	26.8292683	
Wheat	7	5	2.4	0.6	25	
Wheat	7	6	2.9	0.1	2.94117647	
Bamboo	8	1	2.8	0.2	10	24.9871385
Bamboo	8	2	2.2	0.8	25.8064516	
Bamboo	8	3	2.4	0.6	25	
Bamboo	8	4	2.3	0.7	21.875	

Bamboo	8	5	2.5	0.5	17.2413793	
Bamboo	8	6	2.2	0.8	50	
Control	8	1	2.6	0.4	20	23.6128105
Control	8	2	1.7	1.3	41.9354839	
Control	8	3	2.7	0.3	12.5	
Control	8	4	1.6	1.4	43.75	
Control	8	5	2.5	0.5	17.2413793	
Control	8	6	2.9	0.1	6.25	
Mixed	8	1	2.9	0.1	5	20.196476
Mixed	8	2	2.7	0.3	9.67741936	
Mixed	8	3	2.2	0.8	33.3333333	
Mixed	8	4	2.5	0.5	15.625	
Mixed	8	5	2.6	0.4	13.7931035	
Mixed	8	6	2.3	0.7	43.75	
Pine	8	1	1.9	1.1	55	23.9548959
Pine	8	2	2.6	0.4	12.9032258	
Pine	8	3	2.6	0.4	16.6666667	
Pine	8	4	2.7	0.3	9.375	
Pine	8	5	2.1	0.9	31.0344828	
Pine	8	6	2.7	0.3	18.75	
Wheat	8	1	2.8	0.2	10	11.4153458
Wheat	8	2	2.7	0.3	9.67741936	
Wheat	8	3	2.7	0.3	12.5	
Wheat	8	4	2.7	0.3	9.375	
Wheat	8	5	2.4	0.6	20.6896552	
Wheat	8	6	2.9	0.1	6.25	
Bamboo	9	1	2.2	0.8	34.7826087	14.9679826
Bamboo	9	2	2.5	0.5	13.1578947	
Bamboo	9	3	2.8	0.2	4.54545455	
Bamboo	9	4	2.3	0.7	25.9259259	
Bamboo	9	5	2.8	0.2	7.69230769	
Bamboo	9	6	2.9	0.1	3.7037037	
Control	9	1	2.9	0.1	4.34782609	11.687562
Control	9	2	2.6	0.4	10.5263158	
Control	9	3	2.7	0.3	6.81818182	
Control	9	4	2	1	37.037037	
Control	9	5	2.8	0.2	7.69230769	
Control	9	6	2.9	0.1	3.7037037	
Mixed	9	1	2.4	0.6	26.0869565	29.0481582
Mixed	9	2	1.5	1.5	39.4736842	
Mixed	9	3	1.4	1.6	36.3636364	
Mixed	9	4	2.8	0.2	7.40740741	
Mixed	9	5	1.6	1.4	53.8461539	

Mixed	9	6	2.7	0.3	11.1111111	
Pine	9	1	2.6	0.4	17.3913044	20.6850527
Pine	9	2	2.6	0.4	10.5263158	
Pine	9	3	1.4	1.6	36.3636364	
Pine	9	4	2.8	0.2	7.40740741	
Pine	9	5	2.6	0.4	15.3846154	
Pine	9	6	2	1	37.037037	
Wheat	9	1	2.6	0.4	17.3913044	16.2038371
Wheat	9	2	2	1	26.3157895	
Wheat	9	3	2.3	0.7	15.9090909	
Wheat	9	4	2.4	0.6	22.2222222	
Wheat	9	5	2.6	0.4	15.3846154	
Wheat	9	6	3	0	0	
Bamboo	10	1	2.9	0.1	5.88235294	18.2825554
Bamboo	10	2	0.8	2.2	56.4102564	
Bamboo	10	3	2.7	0.3	10	
Bamboo	10	4	2	1	18.8679245	
Bamboo	10	5	2.5	0.5	12.8205128	
Bamboo	10	6	2.8	0.2	5.71428571	
Control	10	1	2.4	0.6	35.2941177	12.5707903
Control	10	2	2.9	0.1	2.56410256	
Control	10	3	2.5	0.5	16.6666667	
Control	10	4	2.3	0.7	13.2075472	
Control	10	5	2.7	0.3	7.69230769	
Control	10	6	3	0	0	
Mixed	10	1	2.6	0.4	23.5294118	22.9576688
Mixed	10	2	2.7	0.3	7.69230769	
Mixed	10	3	1.8	1.2	40	
Mixed	10	4	1.8	1.2	22.6415094	
Mixed	10	5	1.4	1.6	41.025641	
Mixed	10	6	2.9	0.1	2.85714286	
Pine	10	1	2.8	0.2	11.7647059	14.3024573
Pine	10	2	2.3	0.7	17.948718	
Pine	10	3	2.5	0.5	16.6666667	
Pine	10	4	2.3	0.7	13.2075472	
Pine	10	5	2.2	0.8	20.5128205	
Pine	10	6	2.8	0.2	5.71428571	
Wheat	10	1	2.6	0.4	23.5294118	17.6008139
Wheat	10	2	2.4	0.6	15.3846154	
Wheat	10	3	2.5	0.5	16.6666667	
Wheat	10	4	1.3	1.7	32.0754717	
Wheat	10	5	2.3	0.7	17.948718	
Wheat	10	6	3	0	0	
