# Supplementary material for: Potential Role of Masting by Introduced Bamboos in Deer Mice (Peromyscus maniculatus) Population Irruptions Holds Public Health Consequences
Source: PLoS One. 2015 Apr 21;10(4):e0124419. doi: 10.1371/journal.pone.0124419 (PMC4405191; doi:10.1371/journal.pone.0124419)
Supplement: S1 Table — All flowering events took place in cultivated patches in the USA. All flowering bamboos formed caryopses with rates from 40–100% in the introduced range including naturalized species, Phyllostachys aurea and Sasa palmata. (DOCX) [file pone.0124419.s004.docx]

| **Species** | **Origin** | **No. of accessions scored** | **No. of accessions with flowering** | **Percent with fruit** |
| --- | --- | --- | --- | --- |
| Arundinaria gigantea | USA | 45 | 28 | 62 |
| Pleioblastus simonii | Japan | 1 | 1 | 100 |
| Pleioblastus hindsii | China | 3 | 1 | 33 |
| Bambusa textilis | China | 1 | 1 | 100 |
| Phyllostachys aurea | China | 14 | 12 | 86 |
| Phyllostachys bambusoides | China | 88 | 37 | 42 |
| Sasa palmata | Japan, Korea, Russia | 8 | 4 | 50 |

Table S1. Flowering and fruiting from bamboo accessions at the National Herbarium (US).
