# Supplementary material for: Potential Role of Masting by Introduced Bamboos in Deer Mice (Peromyscus maniculatus) Population Irruptions Holds Public Health Consequences
Source: PLoS One. 2015 Apr 21;10(4):e0124419. doi: 10.1371/journal.pone.0124419 (PMC4405191; doi:10.1371/journal.pone.0124419)
Supplement: S2 Table — Calculations, derivations and r-code for the maximum seed attack rate (a) and the seed density at attack rate half saturation (h). (DOCX) [file pone.0124419.s005.docx]

Table S2. Sources for calculating *a:*

Loehman RA, Elias J, Douglass RJ, Kuenzi AJ, Mills JN, Wagoner K. Prediction of *Peromyscus maniculatus* (deer mouse) population dynamics in Montana, USA, using satellite-driven vegetation productivity and weather data. J Wildl Dis. 2012;48: 348–360.

Dalquest, Walter W. 1941. Ecologic relationships of four small mammals in western Washington. Journal of Mammalogy. 22(2): 170-173. [25529]

| Values For Calculation Parameters | | Value |  |
| --- | --- | --- | --- |
| A: Deer mice daily energy consumption | | 48 | kJ/day |
| B: Seed energy at saturation density | | 7.35 x 10^6^ | kJ/ha |
| C: Maximum reported mouse density | | 200 | mice/ha |
| Weekly attack rate = | (A*C/B)*7 | 0.009 | kJ/ha/week |

R Code for calculating H:

#Half saturation density as a function of population plateau duration T

#Duration is on the order of the time required to reach the half-sat density

H <- function(B0, r, T){B0/exp(T*(r))}

#parameter values (rate in weeks)

r <- 0.25       #other sources of seed removal

B0 = xxxxxxx #initial seed density (kJ/h)(varies by plant)

#half saturation densities required for, resp.,

# plateaus of 4, 8, 12, 18, & 24 months:

H(B0, r, 4*4)

H(B0, r, 8*4)

H(B0, r, 12*4)

H(B0, r, 18*4)

H(B0, r, 24*4)
