# Supplementary material for: Potential Role of Masting by Introduced Bamboos in Deer Mice (Peromyscus maniculatus) Population Irruptions Holds Public Health Consequences
Source: PLoS One. 2015 Apr 21;10(4):e0124419. doi: 10.1371/journal.pone.0124419 (PMC4405191; doi:10.1371/journal.pone.0124419)
Supplement: S3 Table — All measurements are an average (± SE) of three 5-g samples that were ground and combusted in an oxygen bomb calorimeter. (DOCX) [file pone.0124419.s006.docx]

Table S3. Caloric content (per g) for seed types fed to deer mice.

|  |  |  |  |  |  |
| --- | --- | --- | --- | --- | --- |
| Sample | Rep | cal/g | Mean | % Protein | % Carbon |
| *Bambusa distigia* | 1 | 4163 | 4132 | 13.88 | 47.6 |
|  | 2 | 3992 |  | 14.02 | 46.5 |
|  | 3 | 4241 |  | 14.7 | 48.2 |
| *Yushania brevipaniculata* | 1 | 4065 | 4065 | 13.89 | 45.2 |
|  | 2 | 4023 |  | 14.04 | 48.4 |
|  | 3 | 4107 |  | 14.25 | 43.9 |
| *Pseudoregneria spicata* | 1 | 4107 | 4050 | 11.85 | 45.9 |
|  | 2 | 4015 |  | 10.01 | 44.7 |
|  | 3 | 4028 |  | 10.62 | 47.9 |
| *Pinus ponderosa* | 1 | 6531 | 6765 | 14.64 | 60.9 |
|  | 2 | 6903 |  | 16.47 | 66.2 |
|  | 3 | 6862 |  | 17.2 | 64.2 |
| *Balsamorhiza sagittata* | 1 | 5789 | 5845 | 15.67 | 55.3 |
|  | 2 | 5912 |  | 17.54 | 56.7 |
|  | 3 | 5834 |  | 16.45 | 58.3 |
| Harklan Teklad Rodent Diet | 1 | 3300 |  | 16.4 | 66 |
|  |  |  |  |  |  |
|  |  |  |  |  |  |
